# Maternal Obesity Promotes Diabetic Nephropathy in Rodent Offspring

**DOI:** 10.1038/srep27769

**Published:** 2016-06-09

**Authors:** Sarah J. Glastras, Michael Tsang, Rachel Teh, Hui Chen, Rachel T. McGrath, Amgad A. Zaky, Carol A. Pollock, Sonia Saad

**Affiliations:** 1 Department of Medicine, Kolling Institute, University of Sydney, Sydney, Australia; 2 Department of Diabetes, Endocrinology and Metabolism, Royal North Shore Hospital, St Leonards, Australia; 3 School of Life Sciences, Faculty of Science, University of Technology Sydney, Australia

## Abstract

Maternal obesity is known to increase the risk of obesity and diabetes in offspring. Though diabetes is a key risk factor for the development of chronic kidney disease (CKD), the relationship between maternal obesity and CKD has not been clearly defined. In this study, a mouse model of maternal obesity was employed to determine the impact of maternal obesity on development of diabetic nephropathy in offspring. Female C57BL/6 mice were fed high-fat diet (HFD) for six weeks prior to mating, during gestation and lactation. Male offspring were weaned to normal chow diet. At postnatal Week 8, offspring were randomly administered low dose streptozotocin (STZ, 55 mg/kg/day for five days) to induce diabetes. Assessment of renal damage took place at postnatal Week 32. We found that offspring of obese mothers had increased renal fibrosis, inflammation and oxidative stress. Importantly, offspring exposed to maternal obesity had increased susceptibility to renal damage when an additional insult, such as STZ-induced diabetes, was imposed. Specifically, renal inflammation and oxidative stress induced by diabetes was augmented by maternal obesity. Our findings suggest that developmental programming induced by maternal obesity has implications for renal health in offspring. Maternal obesity should be considered a risk factor for CKD.

Diabetic nephropathy is an important complication of diabetes and is the leading cause of end-stage kidney disease worldwide^1^. In type 1 diabetes, the risk of diabetic nephropathy is associated with longer duration of diabetes, poor glycemic control, hypertension and dyslipidemia^2^. Yet, it is poorly understood why, despite multiple risk factors for renal disease, some individuals appear to be unaffected by renal complications while others seem to be highly susceptible to diabetic nephropathy^3^. Moreover, there appear to be less well-defined precipitating factors for diabetic nephropathy. Interestingly, recent studies have suggested that maternal factors impacting foetal exposure *in utero* may contributes to increased risk of kidney disease in adulthood^4^,^5^,^6^. This concept of developmental programming is increasingly understood to have long-lasting consequences on renal health.

Developmental programming due to foetal exposure to maternal obesity significantly increases the risk of metabolic syndrome and its sequelae in offspring^7^,^8^. Metabolic syndrome is a cluster of multiple medical comorbidities underpinned by obesity and insulin resistance^9^. A retrospective case control study of over 1 million person years found a 35% increased risk of mortality and 29% greater risk of cardiovascular death in offspring born to obese mothers compared to non-obese mothers^10^. Both rodent and primate models of maternal high-fat diet (HFD) feeding have identified that offspring of obese mothers are at increased risk of obesity, glucose intolerance and diabetes, hepatic steatosis and endothelial injury^11^,^12^. Though the kidney is a highly vascular organ that is very responsive to hemodynamic, metabolic and inflammatory changes^13^, the link between maternal obesity and CKD has not clearly been made.

Our recent studies have identified that rodent offspring of obese mothers have increased renal damage including fibrosis, inflammatory and oxidative stress changes^14^. As such, exposure to maternal obesity *in utero* may propagate renal dysfunction in offspring through increasing the risk of metabolic syndrome, which can further be compounded by the development of diabetes later in life. Thus, the aim of this study was to determine whether maternal obesity increases diabetic nephropathy in rodent offspring with diabetes. Specifically, we aimed to determine whether maternal obesity: (1) leads to changes in renal physiology in offspring at adulthood and, (2) exacerbates renal damage in diabetic offspring, and importantly explore the mechanisms of these effects.

## Results

### Serum metabolic measures

To confirm the diabetic phenotype of mice treated with STZ, indices of glycaemia were assessed. As expected, at the endpoint of Week 32 fasting glucose levels and HbA1c were significantly higher in offspring treated with STZ compared to controls (p < 0.01, Table 1). Thus, STZ induced diabetes in treated animals. There was no difference in fasting glucose or HbA1c between non-diabetic offspring of lean versus obese mothers. Serum insulin levels were increased in offspring of obese mothers; however they were significantly lowered in the offspring with diabetes, in keeping with STZ treatment-induced pancreatic β-cell depletion. Serum triglycerides and NEFA were higher in offspring of obese mothers at postnatal Week 32. HFD induced hypertriglyceridaemia in the offspring, which was reduced in the presence of diabetes (p < 0.01, Table 1).

### Biometric parameters

Total body weight was reduced in offspring with diabetes at 32 weeks of age, irrespective of maternal weight, although kidney/total body weight was similar in all groups (Table 1). Maternal obesity alone did not have a sustained effect on body weight or fasting glucose levels in chow-fed offspring. However, there was significantly increased retroperitoneal and epididymal fat deposits in offspring of obese mothers (p < 0.01). As expected, fat deposits were reduced in diabetic animals, more so in offspring of the obese mothers (Table 1). Liver size was non-significantly increased by diabetes.

### Renal functional and structural changes

The renal function of offspring from obese mothers was considerably worse in comparison to non-obese mothers, especially when they developed diabetes. Both diabetes and maternal obesity increased serum creatinine (CCT1D vs. CC, HC vs. CC and HCT1D vs. CC, P < 0.05, Table 1). 24 h urinary albumin excretion was significantly increased by maternal obesity independent of the development of diabetes and the 24 h urinary albumin excretion was significantly increased in diabetic mice, regardless of the maternal diet (P < 0.05, Table 1). In addition, diabetic animals had polyuria as evidenced by more than three-fold increase in urine output over 24 h (CCT1D vs. CC and HCT1D vs. CC, P < 0.05, Table 1). There was no significant difference in urine output between offspring of obese mothers compared to control.

**Figure 1:**
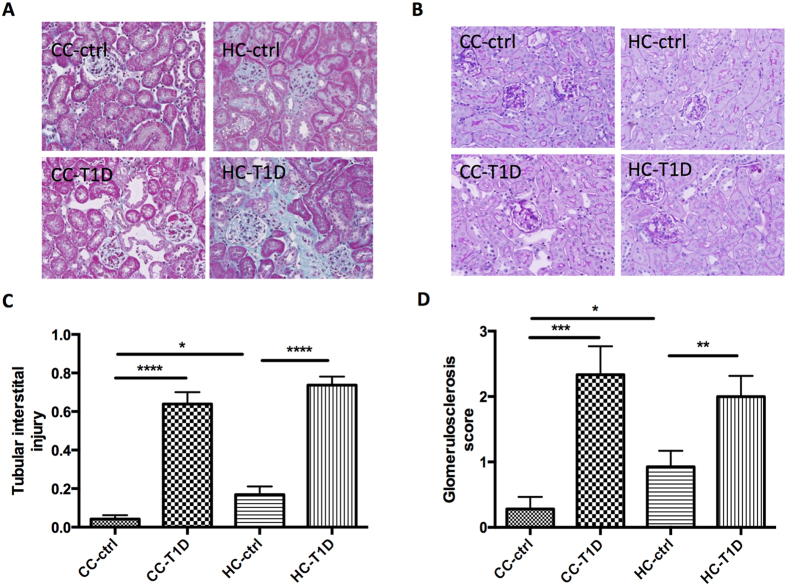
Renal structural changes associated with maternal obesity and diabetes: (**A**) Masson trichrome staining representative image at x20 magnification, (**B**) Periodic acid Schiff staining (PAS) representative image at x20 magnification (**C**) Interstitial fibrosis score assessed by Masson trichrome staining and (**D**) Glomerulosclerosis score assessed by PAS staining. Results are expressed as mean ± SEM, n = 12. *P < 0.05, **P < 0.01, ***P < 0.001, ****P < 0.0001; offspring from lean mothers (control): CC-ctrl, type 1 diabetic offspring from lean mothers: CC-T1D, offspring from obese mothers: HC-ctrl and offspring from obese mothers with type 1 diabetes: HC-T1D.

Objective increases in tubular injury score, determined by Masson trichrome staining, were found in offspring with diabetes regardless of maternal diet (P < 0.01, CC-ctrl vs. CC-T1D; P < 0.01, HC-ctrl vs. HC-T1D) and non-diabetic offspring by maternal obesity (P < 0.05, CC-ctrl vs. HC-ctrl, Fig. 1A,C). Similarly, the glomerulosclerosis score obtained by PAS staining was significantly increased by diabetes (P < 0.01, CC-ctrl vs. CC-T1D; P < 0.01, HC-ctrl vs. HC-T1D) and maternal obesity (P < 0.05 CC-ctrl vs. HC-ctrl, Fig. 1B,D).

### Maternal obesity induced renal fibrosis in the presence of diabetes in offspring

**Figure 2:**
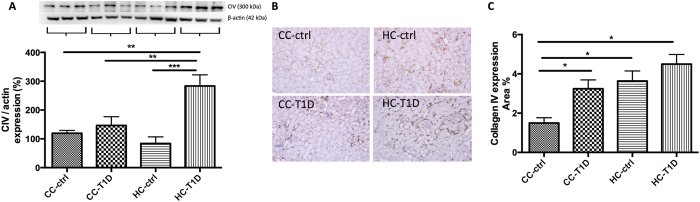
Renal fibrotic changes as measured by Collagen IV: (**A**) Collagen IV protein expression measured by Western blotting (n = 3–5 per group), gels have been run at the same time under the same experimental conditions, (**B**) immunohistochemistry representative image of CIV staining at x20 magnification representative blot (**C**) area % CIV expression (n = 4–6 per group), *P < 0.05, **P < 0.01, ***P < 0.001; offspring from lean mothers (control): CC-ctrl, type 1 diabetic offspring from lean mothers: CC-T1D, offspring from obese mothers: HC-ctrl and offspring from obese mothers with type 1 diabetes: HC-T1D.

**Figure 3:**
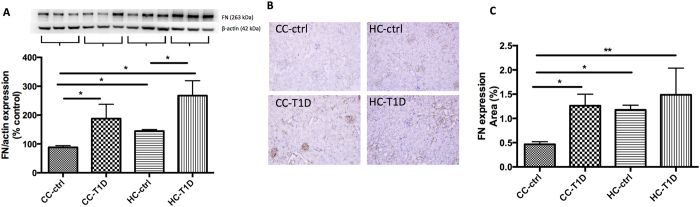
Renal fibrotic changes as measured by fibronectin: (**A**) Fibronectin protein expression by Western blotting (n = 4–6 per group), gels have been run at the same time under the same experimental conditions, (**B**) immunohistochemistry representative image of fibronectin staining at x20 magnification representative blot (n = 4–5 per group), (**C**) area % fibronectin expression. Results are expressed as mean ± SEM, n = 4–6 per group. *P < 0.05, **P < 0.01: offspring from lean mothers (control): CC-ctrl, type 1 diabetic offspring from lean mothers: CC-T1D, offspring from obese mothers: HC-ctrl and offspring from obese mothers with type 1 diabetes: HC-T1D.

To determine whether maternal obesity impacted upon renal fibrosis in offspring, quantitative western blotting and immunohistochemistry were carried out. Protein expression of collagen IV by both Western blotting was increased by maternal obesity in combination with diabetes (P < 0.05, CC-ctrl vs. HC-T1D, P < 0.05, CC-T1D vs. HC- T1D, Fig. 2A). Immunohistochemistry confirmed diabetes increased collagen IV expression compared to control though there was no exacerbating effect of maternal obesity in addition to diabetes (P < 0.05, CC-ctrl vs. HC-T1D, Fig. 2B,C). Likewise, fibronectin protein level measured by western blotting was greater in the diabetes group (P < 0.01, CC-ctrl vs. CC-T1D) and also in mice from obese mothers (P < 0.05, CC-ctrl vs. HC-ctrl, Fig. 3A). There was no significant difference in fibrosis markers when diabetic offspring from lean and obese mothers were compared, suggesting that maternal obesity does not potentiate fibrosis in the presence of diabetes. This finding was confirmed by immunohistochemistry staining (Fig. 3B,C).

### Maternal obesity exacerbates the impact of diabetes on renal inflammation

**Figure 4:**
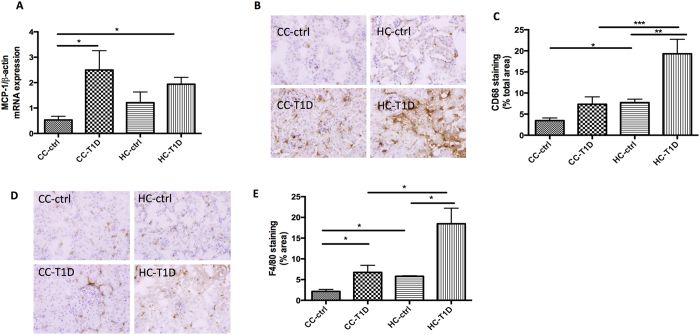
Renal inflammation associated with maternal obesity and diabetes: (**A**) MCP-1 mRNA expression, (**B–C**) CD68 immunohistochemistry staining, and (**D–E**) F4/80 immunohistochemistry staining. Results are expressed as mean ± SEM, n = 6 per group. *P < 0.05; **P < 0.01, ***P < 0.001; offspring from lean mothers (control): CC-ctrl, type 1 diabetic offspring from lean mothers: CC-T1D, offspring from obese mothers: HC-ctrl and offspring from obese mothers with type 1 diabetes: HC-T1D.

Inflammatory changes in the kidney are a hallmark of renal dysfunction in diabetic nephropathy. In keeping with this observation, MCP-1 mRNA expression was greater in the kidneys of offspring with diabetes (P < 0.05, CC-ctrl vs. CC-T1D and HC-T1D, Fig. 4A). Furthermore, levels of the macrophage marker CD68 were significantly increased by diabetes (P < 0.01, HC-ctrl vs. HC- T1D) and maternal obesity (P < 0.05, CC-ctrl vs. CC-T1D, Fig. 4B,C). Similarly, levels of the inflammatory marker F4/80 were higher in the presence of diabetes (P < 0.05, CC-ctrl vs. CC-T1D, P < 0.05, HC-ctrl vs. HC-T1D) and maternal obesity (P < 0.05, CC-ctrl vs. HC-ctrl, Fig. 4D,E). Moreover, the combination of maternal obesity and diabetes gave rise to significantly increased levels of F4/80 compared to diabetes alone (P < 0.01, CC- T1D vs. HC-T1D). Therefore, though maternal obesity in the presence of diabetes had no effect on MCP1 expression, it significantly increased CD68 and F4/80.

### Maternal obesity exacerbates the effect of diabetes on renal oxidative stress

**Figure 5:**
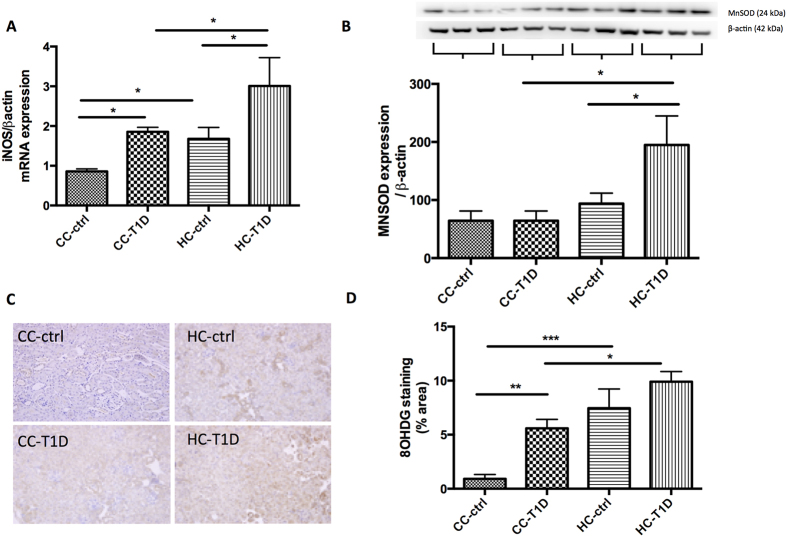
Oxidative stress measures associated with maternal obesity and diabetes: (**A**) iNOS mRNA expression, (**B**) MnSOD protein expression by Western blotting, gels have been run at the same time under the same experimental conditions, and (**C,D**) 8OHdg immunohistochemistry staining. Results are expressed as mean ± SEM, N = 3–6 per group. *P < 0.05; **P < 0.01, ***P < 0.001; offspring from lean mothers (control): CC-ctrl, type 1 diabetic offspring from lean mothers: CC-T1D, offspring from obese mothers: HC-ctrl and offspring from obese mothers with type 1 diabetes: HC-T1D.

Enhanced levels of oxidative stress were observed in the kidneys of offspring of obese mothers, as well as those with diabetes. iNOS mRNA expression was significantly potentiated in offspring of obese mothers with diabetes compared to diabetes alone (P < 0.05, CC-T1D vs. HC-T1D, P < 0.05, HC-ctrl vs. HC-T1D, Fig. 5A). Mitochondrial antioxidant, MnSOD was increased in response to oxidative stress in the kidneys of offspring from obese mothers with diabetes (P < 0.05, CC-T1D vs. HC-T1D, P < 0.05, HC-ctrl vs. HC-T1D, Fig. 5B). Furthermore, immunohistochemistry staining showed 8-OHdg, the major product of DNA oxidation, was significantly increased by diabetes (P < 0.01 CC-ctrl vs. CC-T1D) and maternal obesity (P < 0.001, CC-ctrl vs. HC-ctrl, Fig. 5C,D). Maternal obesity increased 8OH-dg expression. T1D also increased 8-OHdg to similar levels. Interestingly, T1D in the presence of maternal obesity significantly increased 8-OHdg compared to T1D alone (p < 0.01, CC-T1D vs. HC-T1D, Fig. 5C,D).

## Discussion

The results of this study indicate that maternal obesity is associated with renal damage in rodent adult offspring, which is further amplified when diabetes supervenes. Functional and structural changes were seen in the kidneys of diabetic offspring consistent with a model of diabetic nephropathy. Maternal obesity was associated with reduced renal function as measured by serum creatinine and increased renal fibrosis as measured by renal structural changes and fibronectin. Both renal inflammation and oxidative stress were exacerbated in offspring born to obese mothers, further amplified by diabetes, suggesting an important role of developmental programming in the kidney.

The mechanisms underpinning developmental programming of metabolic disease are complex and yet to be fully elucidated, however inflammation and oxidative stress have an integral role in the propagation of metabolic dysfunction between generations^15^,^16^. In addition, there is considerable evidence to support the hypothesis that maternal obesity has significant effects on central pathways for energy homeostatic regulation and reward-driven behavior^17^,^18^,^19^,^20^,^21^,^22^. As a consequence, maternal overnutrition increases adipogenesis and lipogenesis in offspring and leads to adipocyte hyperplasia and hypertrophy due to excessive lipid accumulation, with steatosis occurring in the hepatocytes^23^,^24^,^25^,^26^,^27^,^28^,^29^,^30^. In this study, we found no difference in total adult body weight between offspring of obese versus lean mothers. This is perhaps due to the fact that the offspring were only exposed to a balanced and non-obesogenic diet after weaning. However, the offspring of obese mothers still had increased retroperitoneal and epididymal fat deposits, suggesting body weight does not accurately reflect adiposity. In fact, the concept of normal weight obesity has been proposed, which link this phenomenon to a significantly higher risk of metabolic syndrome, cardiovascular dysfunction and higher mortality rate^31^,^32^. This clearly can be exaggerated by the additional *ad libitum* HFD consumption^21^. However the impact of maternal obesity on kidney pathology in the setting of renal lipid accumulation has not been well studied.

Adipocytes are key secretors of pro-inflammatory cytokines. Indeed, we have previously shown that inflammatory cytokines including IL-6, MCP-1 and TGFβ are increased in the serum of rat offspring of obese mothers, as well as in their kidneys^14^,^33^. Thus, exposure to maternal obesity may prime offspring towards a pro-inflammatory state, thereby increasing their susceptibility to complications of obesity and kidney disease at a later stage. Diabetes itself, even type 1 diabetes in the absence of obesity, is known to foster a pro-inflammatory state and the pathophysiological mechanisms underlying the development of diabetic nephropathy is increasingly being recognized to involve inflammatory processes^34^,^35^. Herein, we report that renal inflammation is exacerbated in the kidneys of diabetic offspring, as a consequence of early exposure to maternal obesity. We found that MCP-1 and macrophage markers CD68 and F4/80 were increased. MCP-1 is an important chemokine, playing a key role in monocyte/macrophage recruitment and contributing to inflammation in diabetic nephropathy^36^,^37^. We further observed that maternal obesity together with diabetes exaggerated inflammatory cell accumulation in the kidney, as seen by increases in both CD68 and F4/80. The inflammatory cells (i.e. macrophages) recruited from the circulation into the interstitium play an active role in tubulointerstitial fibrogenesis^38^, which was also well represented in the offspring’s kidneys at 32 weeks.

Oxidative stress plays a critical role in the pathophysiology of several kidney diseases and has been implicated in the development of diabetic kidney disease in humans and mice^39^,^40^,^41^,^42^. Furthermore, recent studies suggest that production of reactive oxidative species (ROS) in the liver and adipose tissue may precede the onset of foetal weight gain in response to abnormal maternal nutrition *in utero*^17^,^43^. Nutrient excess leads to mitochondrial dysfunction, which in turns gives rise to obesity related pathologies, in part due to the harmful effects of ROS^44^. Mitochondrial dysfunction has been reported as early as embryogenesis in offspring of obese mice, with the oocytes and zygotes having increased mitochondrial membrane potential, higher levels of oxidative phosphorylation and increased ROS production^45^. Mitochondrial DNA (mtDNA) is highly sensitive to the deleterious effects of ROS^46^. The kidney is also highly susceptible to the effects of oxidative stress and mitochondrial dysfunction due to their high metabolic throughput. Indeed, mice with mtDNA mutations die prematurely due to renal failure, suggesting a link between mitochondrial dysfunction and renal disease^47^. To date, no studies have examined the influence of maternal obesity on renal markers of oxidative stress in the context of diabetes. Our findings in this study represent the first evidence of the powerful additive effect of maternal obesity and diabetes on oxidative stress and tissue damage within the kidney. Not only were oxidative stress markers iNOS and 8-OHdg increased but the antioxidant MnSOD was increased; MnSOD is a mitochondrial enzyme that scavenges reactive oxygen species and is known to be upregulated in the context of cellular stress^48^.

In this study we found only mild to moderate glomerulosclerosis and interstitial fibrosis and there was no extensive proteinuria or frank renal sclerosis. Collagen IV expression was variable between western blotting and immunohistochemistry, likely due to the pattern of expression of collagen IV. Specifically, collagen IV is known to be expressed in both the medulla and cortex of the kidney^49^. Therefore, immunohistochemistry analysis better reflects cortical collagen IV expression, known be relevant to renal fibrosis. Our data clearly showed that collagen IV as well as fibronectin werer significantly increased in offspring of obese mothers with diabetes. The Animal Models of Diabetic Complications Consortium have set forth guidelines for the use of animal models of diabetic nephropathy. They have recommended against the usage of C57BL/6 mice as a model of diabetes and its complications due to the relative resistance of the strain to diabetic nephropathy^50^. However, though genetically manipulated animal models such as the eNOS knockout mouse are good models for diabetic nephropathy, they are resistant to the effects of HFD-feeding and this makes them less useful as a model of maternal obesity. Despite the known limitations of the model employed in the present study, there was still significant renal damage in terms of fibrosis in the offspring. This suggests the potent impact of maternal obesity and diabetes to induce renal disorders that can ultimately lead to the development of CKD.

In summary, maternal obesity increases fibrotic, inflammatory and oxidative stress changes in the kidneys of offspring. As expected, diabetes is associated with renal damage involving similar underlying changes. Maternal obesity potentiates the deleterious effects of insulin-insufficient diabetes on offspring kidney health, particularly renal inflammation and oxidative stress. Thus, maternal weight in pregnancy is an important modifiable risk factor for the development of renal disease in offspring later in life.

## Methods

### Animal experiments

**Figure 6:**
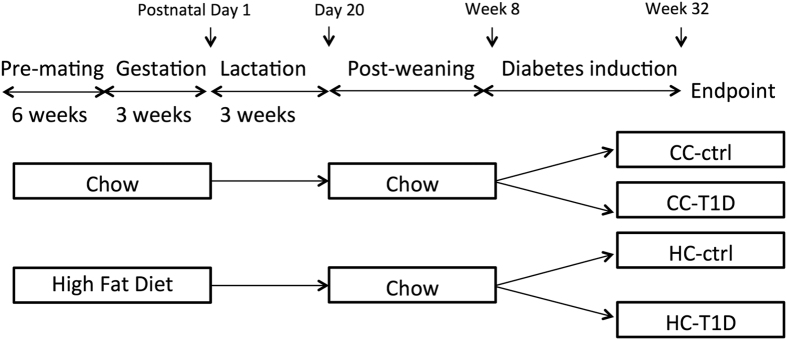
Schematic representation of the mouse model of maternal obesity and offspring diabetes. Offspring from lean mothers (control): CC-ctrl, type 1 diabetic offspring from lean mothers: CC-T1D, offspring from obese mothers: HC-ctrl and offspring from obese mothers with type 1 diabetes: HC-T1D.

A schematic representation of the animal model is shown in Fig. 6. Female C57BL/6 mice were fed either high fat diet (HFD, 20 kJ/g, 43% fat; SF03-020, Specialty Feeds, WA, Australia)) or standard rodent chow (11 kJ/g, 14% fat, Gordon’s Specialty Stockfeeds, NSW, Australia) for six weeks prior to mating (n = 12). They were mated for 24 hours, pregnancy confirmed by vaginal plug and then maintained on the same diet throughout gestation and lactation. Litters were adjusted to 4–6 pups at postnatal Day 1. Male pups were weaned at postnatal day 20 (normal weaning age) and fed chow. At postnatal week 8, mice were assigned to receive either low-dose streptozotocin (STZ) or placebo. Mice receiving low-dose STZ were fasted for 5 h prior to injection. STZ (55 mg/kg, Sigma, MO, USA) was prepared fresh in 0.1 M citrate buffer, pH 4.5 and administered over 5 consecutive days. Animals in the control group were administered citrate buffer.

After fasting for 6 h, mice were weighed and their blood glucose levels were measured fortnightly using an Accuchek glucometer (Roche Diagnostics). Animals with blood glucose >16 mmol/L were considered diabetic. Diabetic mice received insulin treatment (Glargine, Sanofi, Germany) to control ketosis if their blood glucose level was >25 mmol/L; following insulin treatment, their blood glucose readings were performed twice weekly.

Mice were placed in metabolic cages and 24-h urine collection was performed one week prior to animal sacrifice. Urine albumin levels were determined using the Murine Microalbuminuria ELISA kit (Exocell, Inc., Philadelphia, PA, USA).

Animal sacrifice took place at Week 32 under fasting conditions. The body was perfused with PBS, and the kidneys, liver, pancreas and fat were weighed and collected, either snapped frozen for RNA and protein quantification or fixed in 10% buffered formalin for histological examination.

All animals were housed in the Kearns Animal Facility of Kolling Institute, Royal North Shore Hospital and maintained at 22 ± 1 °C with a 12/12-h light–dark cycle. All procedures were approved by the Animal Ethics Committee (AEC) of Royal North Shore Hospital (AEC 1309-007A) and complied with the Australian Code of Practice for the Care and Use of Animals for Scientific Purposes.

### Serum measures

Glycosylated hemoglobin (HbA1c) was measured using a DCA Vantage Analyzer (Siemens Medical Solutions Diagnostics, Tarrytown, NY). To measure serum triglycerides, plasma samples as well as glycerol standards (Sigma-Aldrich, St Louis, MO, USA) were incubated with triacylglycerol reagent (Roche Diagnostics) and concentration was determined using the colorimetric method on a Bio-Rad 680 XR (Hercules, CA, USA). Plasma non-esterified fatty acid (NEFA) was measured using a NEFA kit (WAKO, Osaka, Japan). Serum creatinine was measured using the Architect C16000 Clinical Chemistry Analyzer (Abbott Laboratories, Abbott Park, Ill, USA).

### Analysis of renal structural changes

Formalin-fixed hemisected kidneys were embedded in paraffin and stained with Masson’s trichrome or Periodic Acid Schiff (PAS). They were examined using a light microscope (Leica photomicroscope linked to a DFC 480 digital camera) and five non-overlapping fields were captured for each kidney. Tubular interstitial injury was an aggregate measure of tubular dilatation, separation, atrophy and interstitial fibrosis as measured by a blinded independent nephrologist. Images were analyzed using an Olympus microscope (Olympus, Japan) and Image J software.

### Protein extraction and western blotting

In order to extract protein, 10 mg kidney tissue was washed with phosphate buffered saline (PBS, pH 7.4, containing 0.16 mg/ml heparin to remove any red blood cells and clots) then the tissue was extracted with a Qiagen TissueRuptur (Qiagen, Limburg, Netherlands) in 1.5 ml of cold 20 mM HEPES buffer (pH 7.2, containing 1 mM EGTA, 210 mM mannitol, 70 mM sucrose) and quantified (BioRad, CA, USA). A total of 20 μg of protein sample was loaded onto a NuPAGE SDS-PAGE gel (Life Technologies, CA, USA) and electroblotted to Hybond Nitrocellulose membranes (Amersham Pharmacia Biotech, Bucks, UK). The membranes were incubated overnight at 4 °C with the following primary antibodies: fibronectin (dilution 1:1000, Abcam, Cambridge, UK), collagen IV (1:1000, Abcam), and MnSOD (1:1000, Merck Millipore Ltd., Darmstadt, Germany), followed by horseradish peroxidase conjugated secondary antibody (Life Technologies, CA, USA). Proteins were visualized using Luminata Western HRP Substrate (Millipore, MA, USA) in a LAS 4000 image reader (Fujifilm, Tokyo, Japan). All membranes were re-probed with β-actin 1:1000 (Santa Cruz, CA, USA) and results were expressed as percentage of protein expression relative to β-actin. Analysis was performed using Image J software (Java based software program, National Institutes of Health).

### Immunohistochemistry

Formalin-fixed paraffin kidney sections were deparaffinised and incubated overnight with the polyclonal primary antibody against fibronectin (dilution 1:1,000, Abcam Ltd, Cambridge), collagen IV (dilution 1:1,000, Abcam), CD68 (1:100, ABD Serotec, USA), F4/80 (1:100, ABD Serotec), 8OHdg (1:200, Cell Signalling Ltd) followed by horseradish peroxidase anti-rabbit Envision system (Dako Cytochemistry, Tokyo, Japan) the following day. Staining was developed with 3.3diaminobenzidine tetrahydrochloride (Dako Cytochemistry, Tokyo, Japan) before counterstaining with Mayer’s haematoxylin stains. Antibody against rabbit IgG was used as a negative control. Images were analyzed as described above.

### Relative quantitative real-time PCR (RT-PCR)

RNA was extracted using RNeasy mini kit (Qiagen, Valencia, CA, USA). cDNA was generated using Transcriptor First Strand cDNA Synthesis Kits (Roche Diagnostics, Mannheim, Germany). RT-PCR was performed using an ABI Prism 7900 HT Sequence Detection System (Applied Biosystems, Foster City, CA, USA). SYBR GreenER qPCR Supermix (Invitrogen) with PCR primers was used (Table 2). Results are presented as fold change compared to control after normalization to β-Actin.

### Statistical methods

All results are expressed as mean ± SEM. Data were analyzed using analysis of variance (ANOVA), followed by post hoc Bonferroni tests using GraphPad Prism 6.0 (GraphPad Software, San Diego, CA, USA). A P value of <0.05 was considered statistically significant.

## Additional Information

**How to cite this article**: Glastras, S. J. *et al*. Maternal Obesity Promotes Diabetic Nephropathy in Rodent Offspring. *Sci. Rep*. **6**, 27769; doi: 10.1038/srep27769 (2016).

## Figures and Tables

**Table 1:** Body/metabolic parameters at 32 weeks of age (n = 6–12), *P < 0.05, **P < 0.01, compared with non-diabetic littermates.

	CC-ctrl	CC-T1D	HC-ctrl	HC-T1D
Body weight (g)	26.97 ± 0.37	23.79 ± 0.62**	26.84 ± 0.83	24.20 ± 0.54**
Kidney/Body (% total)	0.85 ± 0.39	0.80 ± 0.74	0.76 ± 0.70	0.75 ± 0.53
Liver/Body (% total)	5.42 ± 0.24	5.48 ± 0.12	5.35 ± 0.19	5.88 ± 0.19
Epididymal mass (% total)	1.59 ± 0.11	1.48 ± 0.15	2.12 ± 0.20#	1.33 ± 0.13**
Retroperitoneal fat (% total)	0.54 ± 0.05	0.46 ± 0.06	0.81 ± 0.07##	0.39 ± 0.05**
Glucose (mmol/L)	14.11 ± 0.46	22.23 ± 0.97**	15.43 ± 0.51	20.28 ± 1.10**
HbA1c (%)	4.51 ± 0.06	5.85 ± 0.24**	4.36 ± 0.11	5.65 ± 0.25**
HbA1c (mmol/mol)	25.76 ± 0.82	40.44 ± 2.97 **	24.15 ± 2.00	38.21 ± 3.02 **
Serum insulin (pmol/L)	67.72 ± 1.97	8.46 ± 3.10**	21.10 ± 5.10#	4.57 ± 2.30**
Serum NEFA (mmol/L)	0.81 ± 0.06	1.06 ± 0.10	1.06 ± 0.23#	1.66 ± 0.18**
Serum triglycerides (mmol/L)	0.25 ± 5.54	0.67 ± 2.01*	0.93 ± 3.49***##	0.43 ± 0.72**
Serum Creatinine (μmol/L)	24.00 ± 0.55	28.67 ± 1.28*	28.00 ± 0.72*	28.00 ± 1.844*
Urine volume (mL/24 h)	0.50 ± 0.11	1.67 ± 0.57*	0.89 ± 0.16	1.62 ± 0.34*
24 h urinary albumin (mg/24 h)	7.71 ± 1.87	31.01 ± 7.00*	30.07 ± 3.87#	42.66 ± 7.55 *

^#^P < 0.05, ^##^P < 0.01. Compared with the control mice with the same treatment, results are expressed as mean ± SEM, n = 6–12.

**Table 2:** Mouse specific primers used in quantitative real time PCR.

Gene	Forward or Reverse	Primer Sequence
MCP	F	5′CTTCTGGGCCTGCTGTTCA
	R	5′CTTCTGGGCCTGCTGTTCA
iNOS	F	5′GACGAGACGGATAGGCAGAG3′
	R	5′GTGGGGTTGTTGCTGAACTT3′
β-Actin (Housekeeping gene)	F	5′-CAAAGCAAAGGCGAGG
	R	5′-ACGGAGCGAAACTGGC
